# 916. The Transfer of Maternal Antibodies and Dynamics of Maternal and Natural Infection-induced Antibodies against RSV in Children: A Longitudinal Cohort Study

**DOI:** 10.1093/ofid/ofad500.961

**Published:** 2023-11-27

**Authors:** Qianli Wang, Meng Xu, Yan Wang, Hongjie Yu

**Affiliations:** Fudan University, Shanghai, Shanghai, China (People's Republic); Fudan University, Shanghai, Shanghai, China (People's Republic); Fudan University, Shanghai, Shanghai, China (People's Republic); Fudan University, Shanghai, Shanghai, China (People's Republic)

## Abstract

**Background:**

Respiratory syncytial virus (RSV) is a leading cause of lower respiratory tract infectious disease in children globally. Transplacental-acquired antibodies are essential in protecting infants during the initial months of their life. This study assessed the efficacy of transferring maternal RSV prefusion protein (pre-F) IgG antibodies via the placenta and reported the dynamics of maternal and natural infection-induced antibodies in children.

**Methods:**

A longitudinal mother-neonate pairs cohort was started enrolled in southern China in the autumn of 2013. 660 mother-neonate pairs were included in this study. Both cord blood from neonates and venous blood from mothers at delivery were obtained. The children were followed up and blood samples were collected at ages 2, 4, 6, 12, 24, and 36 months, as well as at 6-8 years (Figure 1). RSV-specific pre-F IgG antibodies were identified by the enzyme-linked immunosorbent assay method. The maternal antibody transfer efficacy, decay of maternal antibodies, age-specific antibody level, and seroprevalence were estimated.

**Figure:**
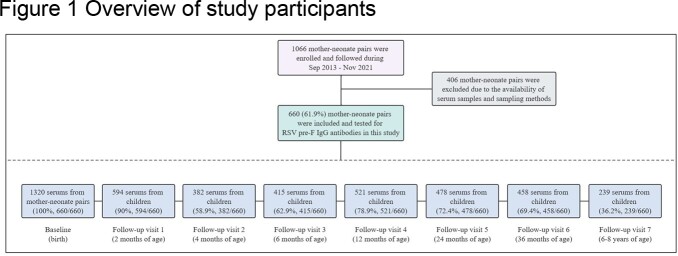

**Results:**

All neonates acquired RSV pre-F IgG antibodies from their mothers. We found a significant correlation between maternal and neonatal antibody titers (ρ=0.86, P< 0.001). The average RSV pre-F IgG antibody titers were higher in cord blood than in maternal blood, with a mean transfer ratio of maternal antibodies of 1.48 (95%CI: 1.44-1.52) (Figure 2). Maternal antibody levels in neonates declined rapidly, with a half-life of 1.4 months. The lowest point of antibodies occurred at 11 months of age, followed by titer increases due to natural RSV infection, and approximately 50% of the children seroconverted at the age of 41.5 months.

**Figure 2:**
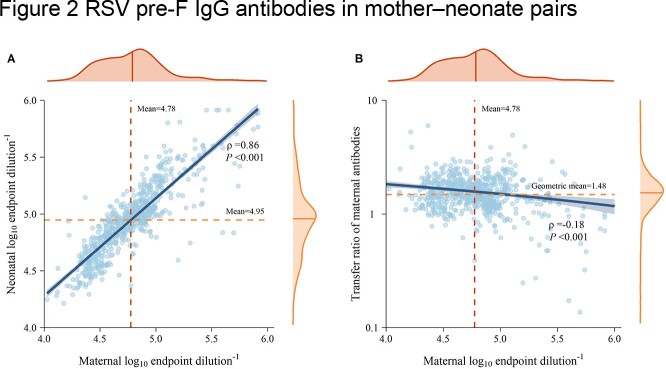
(A) Correlation between neonatal and maternal antibody titer. (B) Transfer ratio with maternal antibody titers. The top orange density plots maternal antibody titers, and the right plots neonatal antibody titers.

**Conclusion:**

Efficient transfer of RSV maternal antibodies to neonates was observed. As maternal antibodies wane, the seropositive infants gradually reduce to the lowest in the first year of life. After that, the infant’s immune system develops and naturally acquired immunity due to infection. Our findings will optimize future strategies for both vaccine and long-term prophylactic monoclonal antibodies to target RSV pre-F protein.

**Disclosures:**

**Hongjie Yu, PhD**, GlaxoSmithKline: Grant/Research Support|Roche: Grant/Research Support|Sanofi: Grant/Research Support|SINOVAC: Grant/Research Support

